# The physical activity at work (PAW) study: a cluster randomised trial of a multicomponent short-break intervention to reduce sitting time and increase physical activity among office workers in Thailand

**DOI:** 10.1016/j.lansea.2022.100086

**Published:** 2022-10-19

**Authors:** Katika Akksilp, Jemima Jia En Koh, Vanessa Tan, Eunice Huiying Tong, Nuttakarn Budtarad, Guo Xueying, Anna Valeria Dieterich, Bee Choo Tai, Andre Matthias Müller, Wanrudee Isaranuwatchai, Thomas Rouyard, Ryota Nakamura, Falk Müller-Riemenschneider, Yot Teerawattananon, Cynthia Chen

**Affiliations:** a Saw Swee Hock School of Public Health, National University of Singapore and National University Health System, Singapore; b Health Intervention and Technology Assessment Programme (HITAP), Ministry of Public Health, Thailand; c Hitotsubashi Institute for Advanced Study, Hitotsubashi University, Japan; d Yong Loo Lin School of Medicine, National University of Singapore and National University Health System, Singapore

**Keywords:** Sedentary behaviour, Physical activity, Workplace, Randomised controlled trial

## Abstract

**Background:**

Sedentary behaviour increases the risks of non-communicable diseases. The objective of this trial was to evaluate the effect of the Physical Activity at Work multicomponent intervention to reduce sedentary behaviour in Thai office workers.

**Methods:**

Offices under the Ministry of Public Health Thailand, were randomly allocated to the intervention and control group in a 1:1 ratio, stratified by office size. The intervention included individual (pedometer and lottery-based financial incentives), social (group movement breaks), environmental (posters), and organisational (leader encouragement) components. At baseline and 6-month follow-up, participants wore ActiGraph^TM^ on the waist for ten days. The primary outcome was the between-group difference in sedentary time at 6-month, analysed using a linear mixed-effects model. Other outcomes were physical activity, biomarkers, productivity, and musculoskeletal health. Trial registration: The PAW study was registered at the Thai Clinical Trials Registry (ID TCTR20200604007) on 02 June 2020.

**Findings:**

282 office workers were recruited and randomly allocated to the control group (142 participants, nine offices) and the intervention group (140 participants, nine offices). The mean age was 38.6 years (SD = 10.4), and 81% were women. There was no evidence of intervention effects on sedentary time during waking hours (−26.8; 95% CI = −69.2 to 15.7 min), physical activity levels, or biomarkers between groups at 6-month. In the adjusted analysis, increases in time spent in moderate-to-vigorous physical activity (5.45; 95% CI = −0.15 to 11.1 min) and step count (718; 95% CI = −45 to 1481 steps) during waking hours were observed, although there was no evidence of a difference between groups.

**Interpretation:**

The intervention did not significantly reduce sedentary time in Thai office workers. Suboptimal intervention uptake due to Covid-19 pandemic restrictions and loss of statistical power associated with recruitment constraints may explain this result. Further investigations are needed to evaluate the processes of the trial.

**Funding:**

The Thai Health Promotion Foundation and the International Decision Support Initiative (iDSI).

**Research in context**

**Evidence before this study**

In parallel with the trial, we conduct a review of systematic reviews of randomised controlled trials to reduce sedentary behaviour in the workplace. Searches were conducted in six databases: Cochrane Systematic Review Database, Cumulative Index to Nursing & Allied Health Literature through EBSCOhost, EMBASE, PubMed including MEDLINE, Scopus and Web of Science. A majority of multicomponent-intervention trials provided significant positive results. However, workplace behavioural intervention studies are performed primarily in high-income countries in Oceania, North America, and Europe.

**Added value of this study**

This study is the first full-scale cluster randomised controlled trial to evaluate the effect of a multicomponent-intervention to reduce sedentary behaviour in office workers in Thailand, Southeast Asia. The multicomponent intervention was developed in the context of Thai culture. Short movement breaks intervention was emphasised as the main intervention component. The trial is the first study in Thailand in which objective measurement of sedentary behaviour and physical activity data using a standardised tool was implemented.

**Implications of all the available evidence**

Our study explores effects of workplace behavioural-change intervention in Thai population. The findings are important for behavioural-change policy development to reduce sedentary behaviour and increase physical activity in Thailand. The outcomes also challenge future intervention design to increase the intervention uptake in the midst of the COVID-19 pandemic.

Alt-text: Unlabelled box

## Introduction

Rapid industrialisation, technologisation, and urbanisation have led to significant economic growth, increased productivity, and improved living standards. However, this has also led to profound lifestyle changes due to the shift toward more sedentary office-based occupations leading to increased sedentary time and physical inactivity.^1^, ^2^, ^3^ Especially among low- and middle-income countries, urban residents exhibit higher sedentary behaviour and physical inactivity than their rural counterparts.^3^, ^4^, ^5^

Sedentary behaviour (SB) is defined as “waking behaviour characterised by an energy expenditure ≤ 1.5 metabolic equivalents, while in a sitting, reclining, or lying posture”, whilst physical inactivity or insufficient moderate-to-vigorous physical activity (MVPA) is defined as less than 150 minutes of moderate-intensity aerobic physical activity weekly for adults aged between 18 and 64 years.^4^^,^^6^^,^^7^ Both SB and insufficient physical activity are risk factors for all-cause mortality and non-communicable diseases,^6^ such as fatal and non-fatal cardiovascular diseases, type 2 diabetes, and metabolic syndrome.^8^

Globally, non-communicable diseases due to physical inactivity have resulted in an estimated worldwide economic burden of (INT $) 67.5 billion in direct healthcare costs and indirect productivity loss in 2013.^9^ Thailand's total cost due to physical inactivity amounted to an estimated (INT $) 190 million,^9^ and approximately 6.3% of total mortality cases were attributable to physical inactivity.^10^ These economic and social burdens of physical inactivity have attracted increasing attention in Thailand in recent years. To address this growing issue, a Cabinet led by the Thai Ministry of Public Health notably formed the “Thailand Physical Activity Strategy 2018–2030” national plan, which implemented Thailand's first “national steps challenge” in 2020.^11^ At a time when there is a strong political will to offset the detrimental consequences of increased sedentary lifestyles in Thailand, it is urgent to identify cost-effective interventions that can be implemented in a real-life setting to reduce SB and physical inactivity.

As office workers spend a large portion of their time sitting, the World Health Organization proposed that employers implement programmes that promote physical activity and/or reduce SB.^4^ To date, some workplace interventions have shown to effectively reduce SB amongst white-collar workers.^12^, ^13^, ^14^, ^15^, ^16^ A systematic review found that multi-component interventions that target multiple ecological levels produced the greatest reduction in sitting time compared to other intervention strategies such as environmental changes or education alone.^17^ This is in line with the literature highlighting the multi-level determinants of occupational SB and physical activity.^18^^,^^19^ The adoption of evidence-based behavioural change approaches and theories, such as the Health Belief Model,^20^ the Behavioural Change Wheel,^21^ and the Socio-Ecological Model,^22^ are frequently used in research and policy development to increase the efficacy of workplace behavioural interventions.^23^, ^24^, ^25^ However, such interventions have primarily been conducted in high-income Western countries; evidence on their effects in other socio-cultural contexts and populations is scarce.^26^^,^^27^ Due to differences in culture and setting, these studies may not be directly relevant to inform policymaking in Thailand and other low and middle-income countries. While there is growing interest in physical activity research in Thailand and other Southeast Asian countries, field experimental studies using longitudinal data and standardised physical activity and SB measures are still lacking to date in the region.

Hence, the primary aim of the Physical Activity at Work (PAW) trial, a 6-month active intervention cluster randomised controlled trial, was to evaluate the effects of a multicomponent intervention, comprising individual, social, environmental, and organisational-level components, in reducing SB in Thai office workers. Secondary and tertiary outcomes were physical activity levels (time spent in light activity, MVPA, and step count), cardiovascular disease biomarkers, reported work productivity, and musculoskeletal health. Cluster randomisation was used to minimise contamination of the complex intervention.

## Method

This study was reported according to the Consolidated Standards of Reporting Trials (CONSORT) reporting guidelines. The checklist can be found in Appendix, Table S4.

### Study design and participants

This was a two-arm parallel cluster-randomised controlled trial. Inclusion criteria for participants were: 1) had an end date for employment after the study completion date; 2) aged 18 years or older; 3) no physical mobility limitation (e.g., wheelchair-bound); 4) work arrangement was either in the office or at home for at least three days a week; 5) owned a smartphone compatible with Fitbit® application. We excluded employees who had been or would be away for an extended leave of more than two weeks and those who were pregnant at any point during the trial. There were no inclusion/exclusion criteria for clusters, however, offices without an eligible individual participant were excluded.

There were twenty available offices in the Department of Medical Services and the International Health Policy Program of the Ministry of Public Health in Thailand. Two offices were excluded as employees from these offices worked in individually enclosed workspaces or were not based in the office, and hence did not fulfil the eligibility criteria. Office directors from the remaining eighteen offices were approached by the research team for participation in the trial. Once the office directors provided consent for their offices (seven to thirty workers per office) to participate, we invited all eligible office workers (n = 449) to participate in the study. Each office was considered a single cluster in this study.

Data were collected at baseline (two weeks before the start of the intervention) and 6-month (the 23^rd^-24^th^ week of the intervention) using ActiGraph^TM^ accelerometers, interviewer-administered questionnaire, physical examination, and blood collection for biomarker assessment. The 12-month and 18-month data collections were not collected due to the COVID-19 lockdown disrupting all data collection procedures, which was a deviation from the registered protocol. Clinical tests were conducted by an independent certified laboratory. Follow-up measures occurred while COVID-19 pandemic restrictions were in place. Since most participants were working from home at the time, they were given the opportunity to reschedule their follow-up appointment within a seven-day timeframe. Study requirements and procedures were explained to the participants and informed consent was obtained prior to baseline assessment and randomisation.

The protocol for the PAW study was registered in the Thai Clinical Trials Registry (TCTR) under the study ID TCTR20200604007.^28^ The study trial protocol has been published elsewhere.^29^ The study was approved by the Ethical Review Committee for Research in Human Subjects, Ministry of Public Health (ECMOPH) (protocol number: 004-2563) in accordance with the Declaration of Helsinki.

### Intervention

The 6-month multicomponent intervention was developed based on the Social-Ecological Model.^22^ Each level of the model was targeted by at least one component of the intervention: 1) individual-level, 2) social-level, 3) environmental-level, and 4) organisational-level components, as detailed below.

#### Individual-level components

Wearable device with real-time feedback: Participants were given a Fitbit® smartwatch (Inspire HR), a tri-axis accelerometer, worn on the wrist to track their physical activity. Real-time feedback for activities such as total step counts, calories burned, heart rate, and active minutes could be viewed on the smartwatch or through the Fitbit® smartphone application. The application connects the smartwatch to a smartphone via Bluetooth. Participants were requested to wear the smartwatch as frequently as possible throughout the intervention period.

Lottery-based incentives: As a form of financial incentive, participants in the intervention group were eligible for a weekly performance-based lottery. The lottery process is detailed in the section on social-level components below.

#### Social-level components

Team movement breaks: Light-to-moderate-intensity movement breaks of at least four minutes were scheduled four times a day. Two participants from each intervention cluster were selected as movement break leaders and trained by the research team to lead the movement breaks using exercise or dance videos played with songs of participants' choice. Alarm reminders were set within a 60-minute interval at 4 local timings at 9.30 am, 10.30 am, 2.30 pm, and 3.30 pm. Participants who worked from home were invited to join the movement breaks via web conferencing.

Team-based incentives: Each week, participants who participated in at least 70% of the movement breaks (i.e., at least 14 of the 20 movement breaks) were eligible for lottery rewards. The participation was measured using the Fitbit® data and the leaders' reports. Intraday data collected from Fitbit® devices were downloaded through a web application programming interface (API) to obtain minute-by-minute data. Participants were considered compliant if they achieved at least 100 steps during a movement break. One winner was randomly selected among eligible participants each week. We used two different levels of reward to enhance peer effect: the winner would receive 1,000 Baht (US$32) if at least 70% of the participants in his/her cluster (i.e., 7 out of 10 participants) also achieved good compliance, or only 500 Baht (US$16) otherwise.

#### Environmental-level components

Posters: Three types of posters [Additional file 1] containing information on 1) health consequences of SB, 2) physical activities, and 3) examples of exercises and stretching were developed using evidence-based Behaviour Change Techniques (BCT).^30^ Two copies of each type were printed on A2-size posters and displayed in noticeable areas in the intervention offices. These posters aimed to give visual cues and facilitate habit formation.

#### Organisational-level components

Leadership support: Office directors encouraged participants to reduce sedentary time and increase physical activities by sending Line^TM^ messages twice a week. Line^TM^ is a freeware communication application widely used among Thai residents. Weekly lottery rewards were announced to all participants via Line^TM^ and given to the winner in person by the director.

### Study outcomes

The primary outcome was the individual's average daily sedentary time during waking hours and working hours on the workday at 6-month follow-up. In this study, a workday was defined as any day from Monday to Friday excluding public holiday and day of absence due to medical or personal reasons. “Waking hours” were defined as the time participants were awake, while “working hours” were a subset for which participants were awake during their reported working office hours. An individual's working and waking hours were identified from an online lifelog system. Using this system, individuals reported their working hours, as well as their sleeping and waking hours. Activities recorded during weekends and sleeping hours were excluded as this study focused on workday data. A valid workday was defined as having a minimum Actigraph^TM^ (explained below) wear time of ten hours during waking hours.

Secondary outcomes were time spent in light activity, time spent in MVPA, step count, and cardiometabolic biomarkers at 6-month follow-up. Tertiary outcomes were work productivity assessed using the Work Productivity and Activity Impairment Questionnaire (WPAI),^31^ and musculoskeletal health assessed using the Standardised Nordic Questionnaire.^32^ Quality-Adjusted Life Years (QALYs) data will be reported in a follow-up economic evaluation study.

### Physical activity levels

Objective physical activity behaviours were measured using the ActiGraph^TM^ wGT3X-BT tri-axial accelerometer (ActiGraph^TM^, Pensacola, Florida, USA). This widely used and commercially available accelerometer showed high validity in laboratory and free-living conditions.^33^ The accelerometer was initialised at a sample rate of 60Hz. Participants were asked to wear the ActiGraph^TM^ with an elastic belt on their waist, above their right hip, for ten 24-hour days during each data collection period. Participants were instructed to remove the device when bathing or swimming and were required to record any removals.

After each data collection, the research team downloaded raw accelerometer data and converted it into 60-seconds epochs using ActiLife 6 software. These 60-seconds epochs files were analysed using RStudio Version 1.4.1103. Wear time validation and data scoring for physical activities were obtained using the R package “PhysicalActivity”. Wear times were defined using Choi's algorithm^34^ and categorised as wear or non-wear. Physical activities were categorised as sedentary (<151 counts per minute (CPM)), light-intensity (151 to 2689 CPM), moderate-intensity (2690 to 6167 CPM), or vigorous-intensity (> 6167 CPM) using Freedson's cut-points.^35^ Step counts and time spent in different activities were extracted from wear data during the day.

### Questionnaires

Participants completed an interviewer-administered general health questionnaire including 1) Thai National Statistical Office's health survey, which captured sociodemographic data, chronic diseases, recent illness(es), and treatment(s) in the last 12 months^36^; 2) sedentary behaviour and physical activity using the Global Physical Activity Questionnaire (GPAQv2)^37^; 3) work productivity using the Work Productivity and Activity Impairment (WPAI)^31^; 4) quality of life using the EQ-5D-5L questionnaire^38^; and 5) musculoskeletal pain using the Nordic Musculoskeletal questionnaire.^32^ At follow-up, intervention-group participants were asked additional questions about the implementation of the intervention.

### Physical examination

Height (to the nearest 0.1 cm) and weight (to the nearest 0.1 kg) were measured using a Tanita WB-380H Digital Scale. Participants were measured without shoes and in their usual office clothing. Neck, waist, and hip circumferences were measured outside of clothing using a cloth measuring tape. Brachial blood pressure was measured twice in all participants using a Citizen CH-456 Blood Pressure Monitor. Each participant was instructed to rest for five minutes before each blood pressure measurement. The average of two blood pressure measurements was used in the data analysis.

### Blood collection

Certified nurses and laboratory technicians from NHEALTH and Clinical Chemistry, Rajavithi Hospital, performed blood collection and tests at the office buildings. About 15 ml of blood was collected for lipid profile, fasting plasma glucose, Hemoglobin A1C, fasting insulin, serum uric acid, and C-reactive protein. Participants were requested to fast for 10 hours before the blood collection.

### Sample size calculation

Sample size was calculated to detect a between-group difference in sedentary time of 23.3 minutes per day, referred from a similar study.^39^ Parameters used were standard deviation (SD) of 45.5, two-tailed significance level of 5%, 80% power, intra-cluster correlation of 0.05, and a coefficient of variation of 0.52. Since there were 18 offices with a minimum of 7 office workers and a maximum of 40 in these offices, assuming unequal cluster sizes with a mean size of 22 (SD=12) workers, the design effect was 2.36. To detect a difference of 23.3 minutes in sedentary time, 288 participants were needed. Assuming a 20% attrition rate, the final estimated sample size was 360 participants.

### Randomisation and blinding

Random permutation block randomisation with stratification by office size (≤10, 11 – 25, >25) was implemented to randomly allocate the offices to the intervention or control arm based on 1:1 ratio, after baseline data collection and informed consent. The stratification method slightly deviated from the registered protocol (<15, 15 – 20, 21 – 25, 26 – 34, and ≥35 participants) to conform to the actual number of participants in each cluster. Due to the nature of the intervention, participants and researchers involved in the data collection could not be blinded to the randomised allocation. Only the researchers in charge of data analysis were blinded.

### Statistical analysis

Baseline characteristics were summarised with either the mean (and standard deviation) or the frequencies (and percentages) for continuous and categorical variables, respectively. In the main analysis, participants were included if they had at least three valid workdays at the 6-month follow-up. Intention to treat analysis was done using multivariate imputation by chain equation (using R package “mice”) to compare the outcomes with the per-protocol analysis.

A linear mixed-effects model was used to analyse between-group differences for the primary, secondary, and tertiary outcomes using the “lmerTest”^40^ package in R. The primary analysis employed an unadjusted model, with intervention status (intervention or control group) as the fixed effect and the office clusters as the random effect. The adjusted model controlled for the following fixed effects: cluster size, wear times (hours spent wearing ActiGraph^TM^), and the respective baseline value of the outcome of interest (for example, when sedentary time was the outcome, baseline sedentary time was adjusted). Random intercepts accounting for office clusters were modelled with an identity covariance structure. All statistical analyses were performed using RStudio Version 4.0.3, and evaluated assuming a two-sided level of significance of 5%.

### Ethics approval and consent to participate

The study has been approved by the Ethical Review Committee for Research in Human Subjects, Ministry of Public Health (ECMOPH), Thailand (IRB00001629). Any modification to the approved protocol will be submitted for a review by the ethics committee. All participants provided written consent prior to the participation.

### Role of funding source

The funders had no role in study design, data collection or analysis, preparation of the manuscript or decision to publish.

## Results

**Figure 1:**
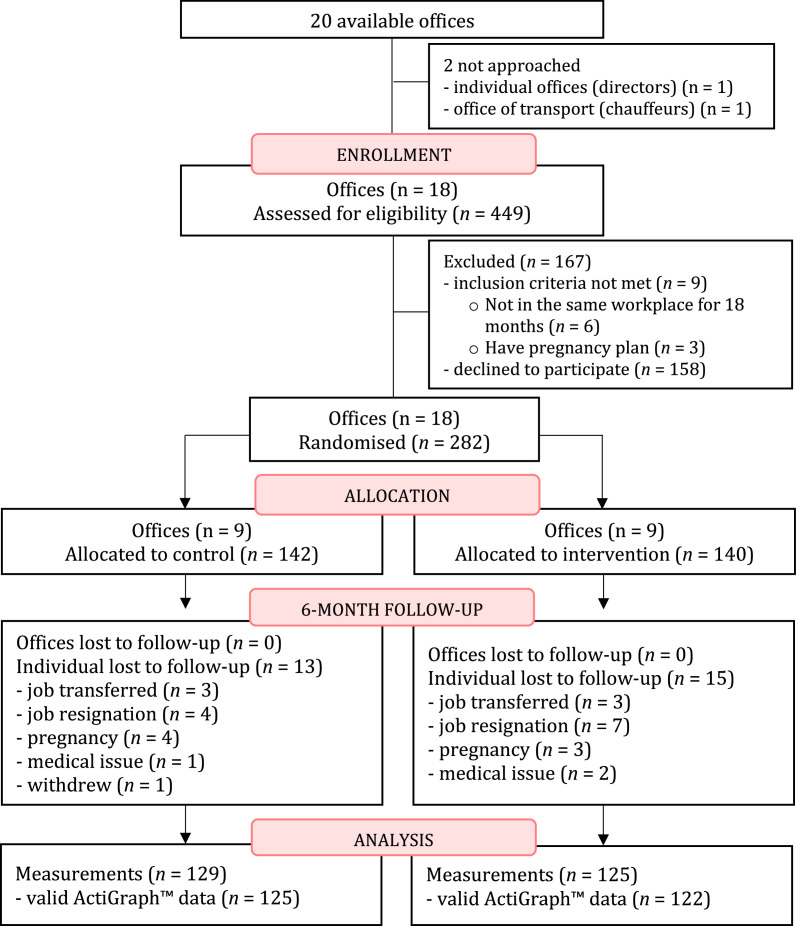
Flow diagram.

Eighteen out of twenty offices from the Ministry of Public Health Thailand, were recruited between July and September 2020. Of 449 eligible office workers, 282 (63%) consented to participate in the study. 142 participants (nine offices) were randomly assigned to the control group and 140 participants (nine offices) were assigned to the intervention group via cluster randomization. All baseline data were successfully collected, with the exception of blood samples for seven participants who declined the procedure. At 6-month follow-up, 13 (9%) and 15 (11%) participants dropped out from the control and intervention groups, respectively. In total, data from 247 participants (125 in the control group; 122 in the intervention group) were included in the primary analyses (Figure 1).

### Office and participant characteristics

There were 6 to 34 participants in each cluster with a median office size of 14 (Table 1). The mean age was 38.6 years (SD = 10.4), and 81% were women. Mean body mass index (BMI) was 24.4 kg/m^2^ (SD = 5.2). Baseline mean sedentary times were 492 min (SD = 107) and 481 min (SD = 110) during waking hours, and 275 min (SD = 45.2) and 271 min (SD = 50.0) during working hours, in the control and intervention groups, respectively. Baseline characteristics were similar in the two groups (Table 2).

**Table 1:** Cluster (offices) characteristics.

	All offices^a^	Offices included in the study		
		Overall	Control	Intervention
Number	20	18	9	9
Office size^b^				
≤10	3	2	1	1
11 – 25	10	10	5	5
>25	7	6	3	3
Median number of workers/office (Range)	25 (4-40)	25 (8-40)	25 (8-40)	25 (11-37)
Participants				
≤10		6	3	3
11 – 25		10	5	5
>25		2	1	1
Median number of participants/office (Range)	-	14 (6-34)	14 (7-28)	14 (6-34)

a All offices from the Department of Medical Services and International Health Policy Program.

b Office size refers to the number of workers in each office, including non-participants.

**Table 2:** Baseline characteristics.

	Overall	Control	Intervention
	**(N = 282, 18 clusters)**	**(N = 142, 9 clusters)**	**(N = 140, 9 clusters)**
Age	38.6 (10.4)	38.0 (10.8)	39.3 (9.9)
Gender, female	228 (80.9%)	116 (81.7%)	112 (80.0%)
Married	88 (31.2%)	44 (31.0%)	44 (31.4%)
Highest education			
- Above Bachelor's degree	100 (35.4%)	49 (34.5%)	51 (36.4%)
- Bachelor's degree	160 (56.7%)	81 (57.0%)	79 (56.4%)
Smoker	17 (6.0%)	8 (5.6%)	9 (6.4%)
Owns a Smartwatch	105 (37.2%)	58 (40.8%)	47 (33.6%)
Body Mass Index (BMI) (kg/m^2^)	24.4 (5.16)	24.4 (5.72)	24.4 (4.52)
Waist hip ratio	0.84 (0.07)	0.84 (0.07)	0.83 (0.06)
Systolic Blood Pressure (mmHg)	119 (17.4)	120 (16.7)	119 (18.1)
Percentage reduced work productivity	19.5 (24.0)	18.7 (23.9)	20.4 (24.2)
Neck pain	162 (57.4%)	88 (62.0%)	74 (52.9%)
Lower back pain	140 (49.6%)	70 (49.3%)	70 (50.0%)
**Sedentary behaviour and physical activity**^a^	**(N = 277, 18 clusters)**	**(N = 139, 9 clusters)**	**(N = 138, 9 clusters)**
**Waking hours**^b^**:**			
Sedentary behaviour, min	486 (109)	492 (107)	481 (110)
Light physical activity, min	321 (85.1)	320 (85.6)	323 (84.9)
Moderate to vigorous physical activity, min Steps	25.0 (18.0) 10.75 (± 8.300)	25.2 (19.9)	24.8 (15.9)
	5696 (1875)	5623 (1937)	5769 (1815)
**Working hours**^b^**:**			
Sedentary behaviour, min	273 (47.6)	275 (45.2)	271 (50.0)
Light physical activity, min	193 (48.3)	191 (46.0)	195 (50.6)
Moderate to vigorous physical activity, min	10.8 (8.30)	10.6 (8.16)	10.9 (8.47)
Steps	3353 (1086)	3287 (898)	3419 (1246)
**Biomarkers**^c^	**(N = 275, 18 clusters)**	**(N = 140, 9 clusters)**	**(N = 135, 9 clusters)**
Cholesterol (mg/dL)	211 (36.3)	211 (34.7)	212 (37.9)
Triglyceride (mg/dL)	105 (61.7)	103 (53.6)	107 (69.3)
High-density lipoprotein cholesterol (mg/dL)	56.5 (12.4)	55.9 (12.9)	57.0 (11.8)
Low-density lipoprotein cholesterol (mg/dL)	137 (35.3)	138 (35.5)	137 (35.3)
Fasting blood glucose (mmol/L)	90.4 (29.2)	89.8 (25.6)	91.1 (32.6)
Hemoglobin A1C (%)	5.44 (1.28)	5.38 (0.87)	5.50 (1.59)
Fasting insulin (uU/mL)	8.57 (10.0)	8.73 (6.72)	8.40 (12.6)
Uric acid (mg/dL)	5.29 (1.42)	5.31 (1.36)	5.26 (1.48)
C-Reactive Protein (mg/L)	3.09 (5.07)	3.10 (5.58)	3.07 (4.51)

Categorical variables are expressed in count (percentage); Continuous variables are expressed in mean (standard deviation).

a Sedentary behaviour and physical activity data contain smaller samples due to invalid ActiGraph^TM^ data.

b Waking hours and working hours data obtained from participants’ daily log.

c Biomarkers data contain smaller samples due to 7 participants declining blood collection.

During the 6-month intervention period, 480 movement break sessions were delivered to the intervention group. The average (in this case; median) proportion of sessions attended by participants was 31.5% (IQR 20.4% to 42.7%) (Figure S1).

### Intervention effects

The results at 6-month follow-up, including sedentary behaviour, physical activity, health and work-related outcomes are shown in Tables 3 and 4. 247 participants provided valid objective physical activity data with mean valid days of 5.3 days (SD = 1.2), mean wear time during waking hours of 13.8 hours (SD = 2.7), and mean wear time during working hours of 7.9 hours (SD = 0.2), which were similar between the control and intervention groups (Table S1). Per-protocol analysis (125 control and 122 intervention participant data) of the primary outcome showed no evidence of difference in sedentary time between groups during waking hours (mean difference =−26.8 min; 95%CI = −69.2 to 15.7 min) and working hours (mean difference= −3.99 min; 95%CI = −25.0 to 17.0 min) at 6-month follow-up (Table 3). Intention to treat analysis with missing data imputation showed consistent results (Table S2). In addition, there was no evidence of intervention effect on time spent in light physical activity, MVPA, or step count at 6-month follow-up, neither during working nor waking hours. Increases in time spent in MVPA (mean difference = 5.45 min; 95%CI = −0.15 to 11.1 min) and step count (mean difference = 718; 95%CI = −45 to 1481) were observed during waking hours in the adjusted analysis, which were also not statistically significant (Table 4).

**Table 3:** Primary (time spent in sedentary behaviour) outcomes.

	6-month, Mean (SD)		Mean difference (Intervention – control)			Adjusted Mean difference (Intervention – control)		
Outcome	Control (*n* = 125)	Intervention (*n* = 122)	β (95% CI) ^a^	*P*-value	ICC b	β (95% CI) c	*P*-value	ICC b
Waking hours^d^								
Sedentary behaviour, min	496 (114)	474 (125)	−26.8 (−69.2 – 15.7)	0.20	0.04	−9.54 (−35.4 – 16.4)	0.44	0.07
Working hours^d^								
Sedentary behaviour, min	276 (50.4)	273 (52.4)	−3.99 (−25.0 – 17.0)	0.69	0.08	−0.54 (−15.5 – 14.4)	0.94	0.05

^Aa^Linear mixed-effect model (unadjusted), accounting for office cluster as a random effect variable.

b ICC stands for Intra-Cluster Correlations.

c Linear mixed-effect model adjusted for wear time, office size, and the respective baseline value of the outcome of interest.

d Waking hours and working hours data obtained from participants’ daily log.

**Table 4:** Secondary (physical activity and biomarkers) outcomes.

	6-month, Mean (SD)		Mean difference (Intervention – control)		Adjusted Mean difference (Intervention – control)	
Physical Activity	Control (*n* = 125)	Intervention (*n* = 122)	β (95% CI) a	*P*-value	β (95% CI) b	*P*-value
Waking hours						
Light physical activity, min	317 (88.4)	317 (94.6)	-5.31 (-45.5 – 34.8)	0.78	2.28 (-25.2 – 29.7)	0.86
Moderate to vigorous physical activity, min	24.4 (19.3)	29.5 (20.4)	5.26 (-1.35 – 11.9)	0.11	5.45 (-0.15 – 11.1)	0.06
Steps	5237 (2075)	5953 (2395)	743 (-279 – 1766)	0.14	718 (-45 – 1481)	0.06
Working hours^c^						
Light physical activity, min	186 (50.7)	189 (49.7)	4.66 (-20.8 – 30.1)	0.70	-2.37 (-16.5 – 11.7)	0.72
Moderate to vigorous physical activity, min	10.9 (9.01)	14.3 (14.1)	3.60 (-0.85 – 8.05)	0.11	3.28 (-1.68 – 8.25)	0.18
Steps	3059 (1250)	3418 (1680)	455 (-269 – 1180)	0.20	191 (-227 – 608)	0.34
**Physical examination and Biomarkers**	**Control (*n* = 125)**	**Intervention (*n* = 122)**	**β (95% CI)**	***P*-value**	**β (95% CI)**^d^	***P*-value**
Body Mass Index (BMI)	24.1 (5.48)	24.1 (4.30)	0.01 (-1.39 – 1.42)	0.98	-0.22 (-0.61 – 0.16)	0.24
Waist to Hip ratio	0.852 (0.117)	0.845 (0.072)	-0.01 (-0.03 – 0.02)	0.60	0.00 (-0.03 – 0.03)	0.94
Systolic blood pressure (mmHg)	122 (18.0)	124 (17.9)	2.33 (-2.79 – 7.44)	0.34	3.52 (-0.12 – 7.17)	0.06
Pulse (beat per min)	83.2 (10.5)	83.5 (11.1)	0.30 (-2.88 – 3.48)	0.84	-0.32 (-3.54 – 2.90)	0.84
Cholesterol^e^ (mg/dL)	202 (34.7)	203 (33.0)	3.26 (-10.1 – 16.6)	0.61	-0.03 (-6.53 – 6.46)	0.99
Triglyceride^e^ (mg/dL)	102 (55.9)	102 (61.3)	0.10 (-16.8 – 17.0)	0.99	-7.88 (-18.9 – 3.12)	0.15
High-density lipoprotein Cholesterol^e^ (mg/dL)	58.1 (12.9)	59.1 (11.6)	1.29 (-2.67 – 5.25)	0.49	0.49 (-1.43 – 2.41)	0.59
Low-density lipoprotein Cholesterol^e^ (mg/dL)	133 (35.5)	133 (33.9)	1.30 (-9.91 – 12.51)	0.81	0.51 (-5.91 – 6.94)	0.86
Fasting blood sugar^e^ (mmol/L)	85.9 (24.2)	89.0 (33.1)	3.57 (-6.83 – 14.0)	0.47	1.27 (-2.37 – 4.90)	0.47
Hemoglobin A1C e (%)	5.33 (0.781)	5.41 (1.19)	0.10 (-0.26 – 0.46)	0.57	-0.01 (-0.13 – 0.11)	0.86
Fasting Insulin^e^ (uU/mL)	6.84 (4.47)	6.57 (4.41)	-0.27 (-1.54 – 1.01)	0.66	-0.44 (-1.50 – 0.62)	0.38
Uric acid^e^ (mg/dL)	5.34 (1.41)	5.19 (1.40)	-0.16 (-0.56 – 0.25)	0.42	-0.12 (-0.34 – 0.11)	0.28
C-Reactive Protein^e^ (mg/L)	3.00 (7.11)	3.00 (5.39)	0.03 (-1.94 – 2.00)	0.97	0.06 (-1.87 – 1.98)	0.95

a Linear mixed-effect model (unadjusted), accounting for office cluster as a random effect variable.

b Linear mixed-effect model adjusted for wear time, office size, and the respective baseline value of the outcome of interest.

c Waking hours and working hours data obtained from participants’ daily log.

d Linear mixed-effect model adjusted for the respective baseline covariate.

e Biomarkers data contain smaller samples due to refusal to blood collection (control; *n* = 124, intervention; *n* = 119).

There was no statistically significant between-group difference in lipid profile, sugar level, or cardiovascular risk biomarkers. When adjusting for the respective baseline covariate, we found reduced levels of triglyceride mean difference =−7.88 mg/dL; 95% CI = −18.9 to 3.12 mg/dL), and higher systolic blood pressure (mean difference =3.52; 95%CI = −0.12 to 7.17 mmHg) in the intervention group, although not statistically significant (Table 4). We also identified no significant difference in self-reported work productivity between groups. Finally, neck and lower back pain were reported and showed no significant difference between groups (Table S3).

## Discussion

This cluster randomised controlled trial aimed to evaluate the first complex intervention designed to reduce sedentary behaviour in office workers in Southeast Asia. The PAW multicomponent intervention was developed based on the Socio-Ecological model and involved individual, social, organisational, and environmental components.

We found no evidence of intervention effect on sedentary time during waking hours or working hours at 6-month follow-up. These results were in accordance with some previous studies.^41^, ^42^, ^43^, ^44^, ^45^ Our adjusted analysis also showed an increase in time spent in MVPA and step count during waking hours in the intervention arm although without sufficient evidence to support (Table 4).

Other intervention components shown as being promising in previous studies might have increased the effectiveness of PAW. For example, regarding individual components, goal-setting strategies using baseline physical activity information to encourage participants to create their own achievable goals are suggested to be effective.^46^^,^^47^ However, conflicting evidence has also been reported, highlighting variations in effects across settings and populations.^48^ Regarding social-level components, Patel and colleagues found a larger increase in physical activity level in a “competitive gamification” arm than in a “support and collaboration” arm.^47^ At the environmental level, height-adjustable workstations are a potentially effective intervention component to reduce sitting time in the office.^17^^,^^46^^,^^49^, ^50^, ^51^ We did not include such a component in this study due to working space constraints.

Another potential cause of the absence of significant effect in our study may be due to low attendance to movement break sessions (median percentage of participation was 31.5%, IQR = 20.4%–42.7%). Most participants in the intervention group (77.8%) attended less than half of the movement break sessions. One reason could be the work arrangement during the COVID-19 pandemic, which permitted some of the participants to work from home (up to 50% of participants working from home in 16 clusters, and up to 100% in the remaining 2 clusters). Although movement breaks were also encouraged via web conferencing, peer pressure and support were de facto reduced and poor internet connection may have further hindered attendance. Moreover, working from home prevented exposure to the environmental- and organisational-components since posters were in offices and the reward ceremonies were changed from face-to-face to online announcements.

Not only did the Covid-19 pandemic affect intervention uptake, but it also directly increased sedentary time. Indeed, a recent systematic review reported increases in sedentary behaviours during lockdowns^52^ and a meta-analysis showed that sedentary time increased up to 127 min per day in the adult population.^53^ Other studies also found increases in sedentary behaviour due to work from home arrangements.^54^^,^^55^

Finally, the lack of significant effects may reflect an improvement in the health status of participants in both groups. We observed decreases in cholesterol, LDL cholesterol, fasting blood sugar, and C-reactive protein at 6-month follow-up compared to baseline in both groups. Improvement in the self-report neck and lower back pain was also observed. This might be due to the Hawthorne effect as they became more aware of their health as a result of being observed.

### Strength and limitations

The PAW study is a large cluster randomised controlled trial with 6-month follow-up period. The trial protocol was published before the start of the trial. Objective data collection was done using a standard accelerometer with a 10-day wear duration to ensure the accuracy of the average physical activity level captured in the trial^56^ and prevent inaccuracy from self-report bias.^57^ The complex intervention was developed using behavioural change theory^24^ and the Socio-Ecological model^22^ and was piloted in small groups of office workers for a short period. The trial was conducted in a real work setting. Moreover, the intervention components are easy to implement at a low cost in any worksite.

Nevertheless, some limitations need to be acknowledged. First, the actual number of participants in the primary outcome analysis was smaller than the sample size required by our power calculation (247 of 282; 87.6%) due to the low recruitment rate. Therefore, our study may be underpowered to detect a sedentary time difference of 23.3 min per day. Moreover, the observed variability in sedentary time during the waking hours (SD = 119.8) was more than twice the postulated (SD = 45.5) during the trial design stage. This further resulted in underestimation of sample size for detecting intervention effect for this outcome. Second, many study participants had low sedentary time at baseline (working hours mean sedentary time = 273 min). In addition, 105 participants (37.2%) owned a Smartwatch at baseline. This may reflect an already high physical health awareness, thereby limiting the external validity of the findings. Lastly, the intervention was not actually designed for implementation during the pandemic since it was intended for an in-office population.

## Conclusions

In summary, despite sound intervention development processes and robust methodology, the PAW multicomponent intervention programme showed no evidence of sedentary time reduction in Thai office workers. Suboptimal intervention uptake due to Covid-19 pandemic restrictions and loss of statistical power associated with recruitment constraints may explain this result. Further investigations are needed to evaluate the processes of the study.

## Contributors

All authors contributed to the study design and/or delivery of the trial. KA was Principal Investigator (PI) of the trial. KA, CC, AD, JK, VT, and THE drafted the manuscript together. TBC, JK, XY and CC provided statistical expertise, data management and sample size calculation. YT, WI, WR, SJ, and NB provided expertise on economic evaluation and were responsible for conceptualising economic aspects. RN and TR provided expertise on behavioural economics, such as the design of the lottery-based and team-based incentives. FMR and AMM provided expertise on the effects of PA and SB on health, measuring and analysing PA data and designing a behaviourally informed intervention. All authors have reviewed the manuscript draft, have read, and approved the final version.

## Data sharing statement

Participants' data (e.g., case record forms, laboratory test, information sheets, and consents) are stored in a locked cabinet in a researchers' office. All data will be destroyed by researchers within five years after publications. During the study, only de-identified data were used, and the data were only accessible to the research team. The research team will have exclusive rights to the de-identified data for 24 months after the trial is completed. After that, the data and full protocol will be publicly accessible on the HITAP website. Consent from participants were obtained to publish the results from de-identified data.

## Declaration of interests

The authors do not have conflicts of interest to report.

## Appendix Supplementary materials

Image, application 1

Image, application 2

Image, application 3
