# Supplementary material for: The physical activity at work (PAW) study: a cluster randomised trial of a multicomponent short-break intervention to reduce sitting time and increase physical activity among office workers in Thailand
Source: Lancet Reg Health Southeast Asia. 2022 Oct 19;8:100086. doi: 10.1016/j.lansea.2022.100086 (PMC10305858; doi:10.1016/j.lansea.2022.100086)
Supplement: Supplementary file 1 [file mmc1.docx]

Appendix

**Figure S1.** Intervention group participants’ participation to movement breaks intervention


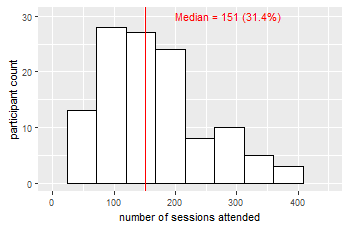


| **Table S1.** Baseline and 6-month ActiGraph™ measurements | | | | | | | |
| --- | --- | --- | --- | --- | --- | --- | --- |
|  |  | Overall | | Control | | Intervention | |
|  |  | N | Mean (SD) | N | Mean (SD) | N | Mean (SD) |
| Number of valid days (days) | Baseline | 277 | 5.3 (1.0) | 139 | 5.2 (0.9) | 138 | 5.2 (0.9) |
|  | 6-month | 247 | 5.3 (1.2) | 125 | 5.1 (0.9) | 122 | 5.6 (1.3) |
| Waking hours ^a^  wear time (h/ day) | Baseline | 277 | 13.9 (2.4) | 139 | 13.9 (2.4) | 138 | 13.8 (2.4) |
|  | 6-month | 247 | 13.8 (2.7) | 125 | 14.0 (2.4) | 122 | 13.7 (2.9) |
| Working hours ^a^  wear time (h/ day) | Baseline | 277 | 7.9 (0.2) | 139 | 7.9 (0.2) | 138 | 8.0 (0.2) |
|  | 6-month | 247 | 7.9 (0.2) | 125 | 7.9 (0.3) | 122 | 7.9 (0.2) |
| ^a^ Waking hours and working hours data obtained from participants’ daily log | | | | | | | |

| **Table S2.** Primary (time spent in sedentary behaviour) outcome with imputation of missing values | | | | | | |
| --- | --- | --- | --- | --- | --- | --- |
|  | 6-month, Mean (SD) | | | Mean difference  (Intervention – control) | | |
| Outcome | Control  (n = 142) | Intervention  (n = 140) | | β (95% CI) ^a^ | P-value | ICC |
| Waking hours ^b^ |  |  |  |  |  |  |
| Sedentary behaviour, min | 499  (111) | 478  (128) | | -26.1  (-66.9 – 14.7) | 0.19 | 0.04 |
| Working hours ^b^ |  |  |  |  |  |  |
| Sedentary behaviour, min | 276  (49.8) | 272  (51.7) | | -4.75  (-24.5 – 15.0) | 0.62 | 0.08 |
| ^a^ Linear mixed-effect model (unadjusted), controlling for clustering  ^b^ Waking hours and working hours data obtained from participants’ daily log | | | | | | |

| **Table S3.** Tertiary outcomes | | | | | | |
| --- | --- | --- | --- | --- | --- | --- |
|  | 6-month,  Mean (SD) | | Mean difference  (Intervention – control) | | Adjusted Mean difference  (Intervention – control) | |
| Outcome | Control  (n = 125) | Intervention  (n = 122) | β (95% CI) ^a^ | P-value | β (95% CI) ^b^ | P-value |
| Percentage reduced work productivity | 21.8  (25.3) | 21.6  (27.0) | 0.25  (-8.53 – 9.02) | 0.95 | -0.37  (-9.20 – 8.47) | 0.93 |
| Musculoskeletal ^c^ complaints |  |  | Odds ratio ^c^  (Intervention – control) | | Adjusted Odds ratio ^c^  (Intervention – control) | |
| Neck pain  (count, percent) | 60  (48%) | 74  (61%) | 0.74  (0.34 – 1.61) | 0.45 | 0.77  (0.33 – 1.83) | 0.56 |
| Lower back pain  (count, percent) | 45  (36%) | 40  (33%) | 0.87  (0.51 – 1.47) | 0.60 | 0.82  (0.46 – 1.44) | 0.48 |
| ^a^ Linear mixed-effect model (unadjusted), controlling for clustering  ^b^ Linear mixed-effect model, adjusted for the respective baseline covariate  ^c^ β is replaced by Odds ratio for dichotomised outcomes | | | | | | |

**Table S4. CONSORT 2010 checklist of information to include when reporting a cluster randomised trial**

| Section/Topic | Item No | Standard Checklist item | Extension for cluster designs | Page No * |
| --- | --- | --- | --- | --- |
| Title and abstract | | | |  |
|  | 1a | Identification as a randomised trial in the title | Identification as a cluster randomised trial in the title | 1 |
|  | 1b | Structured summary of trial design, methods, results, and conclusions (for specific guidance see CONSORT for abstracts)^[[1]](#endnote-1),^^[[2]](#endnote-2)^ | See table 2 | 2 |
| Introduction | | | |  |
| Background and objectives | 2a | Scientific background and explanation of rationale | Rationale for using a cluster design | 5 |
|  | 2b | Specific objectives or hypotheses | Whether objectives pertain to the cluster level, the individual participant level or both | 4 |
| Methods | | | |  |
| Trial design | 3a | Description of trial design (such as parallel, factorial) including allocation ratio | Definition of cluster and description of how the design features apply to the clusters | 5 |
|  | 3b | Important changes to methods after trial commencement (such as eligibility criteria), with reasons |  | 11 |
| Participants | 4a | Eligibility criteria for participants | Eligibility criteria for clusters | 5 |
|  | 4b | Settings and locations where the data were collected |  | 10 |
| Interventions | 5 | The interventions for each group with sufficient details to allow replication, including how and when they were actually administered | Whether interventions pertain to the cluster level, the individual participant level or both | 6-8 |
| Outcomes | 6a | Completely defined pre-specified primary and secondary outcome measures, including how and when they were assessed | Whether outcome measures pertain to the cluster level, the individual participant level or both | 8-9 |
|  | 6b | Any changes to trial outcomes after the trial commenced, with reasons |  | **-** |
| Sample size | 7a | How sample size was determined | Method of calculation, number of clusters(s) (and whether equal or unequal cluster sizes are assumed), cluster size, a coefficient of intracluster correlation (ICC or *k*), and an indication of its uncertainty | 11 |
|  | 7b | When applicable, explanation of any interim analyses and stopping guidelines |  | **-** |
| Randomisation: | | | |  |
| Sequence generation | 8a | Method used to generate the random allocation sequence |  | **11** |
|  | 8b | Type of randomisation; details of any restriction (such as blocking and block size) | Details of stratification or matching if used | 11 |
| Allocation concealment mechanism | 9 | Mechanism used to implement the random allocation sequence (such as sequentially numbered containers), describing any steps taken to conceal the sequence until interventions were assigned | Specification that allocation was based on clusters rather than individuals and whether allocation concealment (if any) was at the cluster level, the individual participant level or both | 11 |
| Implementation | 10 | Who generated the random allocation sequence, who enrolled participants, and who assigned participants to interventions | Replace by 10a, 10b and 10c |  |
|  | 10a |  | Who generated the random allocation sequence, who enrolled clusters, and who assigned clusters to interventions | 11 |
|  | 10b |  | Mechanism by which individual participants were included in clusters for the purposes of the trial (such as complete enumeration, random sampling) | 5 |
|  | 10c |  | From whom consent was sought (representatives of the cluster, or individual cluster members, or both), and whether consent was sought before or after randomisation | 6 |
|  |  |  |  |  |
| Blinding | 11a | If done, who was blinded after assignment to interventions (for example, participants, care providers, those assessing outcomes) and how |  | 11 |
|  | 11b | If relevant, description of the similarity of interventions |  | **-** |
| Statistical methods | 12a | Statistical methods used to compare groups for primary and secondary outcomes | How clustering was taken into account | 11-12 |
|  | 12b | Methods for additional analyses, such as subgroup analyses and adjusted analyses |  | 12 |
| Results | | | |  |
| Participant flow (a diagram is strongly recommended) | 13a | For each group, the numbers of participants who were randomly assigned, received intended treatment, and were analysed for the primary outcome | For each group, the numbers of clusters that were randomly assigned, received intended treatment, and were analysed for the primary outcome | 13 |
|  | 13b | For each group, losses and exclusions after randomisation, together with reasons | For each group, losses and exclusions for both clusters and individual cluster members | 13 |
| Recruitment | 14a | Dates defining the periods of recruitment and follow-up |  | 12 |
|  | 14b | Why the trial ended or was stopped |  | - |
| Baseline data | 15 | A table showing baseline demographic and clinical characteristics for each group | Baseline characteristics for the individual and cluster levels as applicable for each group | 14-15 |
| Numbers analysed | 16 | For each group, number of participants (denominator) included in each analysis and whether the analysis was by original assigned groups | For each group, number of clusters included in each analysis | 12 |
| Outcomes and estimation | 17a | For each primary and secondary outcome, results for each group, and the estimated effect size and its precision (such as 95% confidence interval) | Results at the individual or cluster level as applicable and a coefficient of intracluster correlation (ICC or k) for each primary outcome | 17 |
|  | 17b | For binary outcomes, presentation of both absolute and relative effect sizes is recommended |  | - |
| Ancillary analyses | 18 | Results of any other analyses performed, including subgroup analyses and adjusted analyses, distinguishing pre-specified from exploratory |  | - |
| Harms | 19 | All important harms or unintended effects in each group (for specific guidance see CONSORT for harms^[[3]](#endnote-3)^) |  | - |
| Discussion | | | | 19-21 |
| Limitations | 20 | Trial limitations, addressing sources of potential bias, imprecision, and, if relevant, multiplicity of analyses |  | 21 |
| Generalisability | 21 | Generalisability (external validity, applicability) of the trial findings | Generalisability to clusters and/or individual participants (as relevant) | 20-21 |
| Interpretation | 22 | Interpretation consistent with results, balancing benefits and harms, and considering other relevant evidence |  | **21-22** |
| Other information | | |  |  |
| Registration | 23 | Registration number and name of trial registry |  | 2 |
| Protocol | 24 | Where the full trial protocol can be accessed, if available |  | 6 |
| Funding | 25 | Sources of funding and other support (such as supply of drugs), role of funders |  | 23 |

** Note: page numbers optional depending on journal requir*

1. Hopewell S, Clarke M, Moher D, Wager E, Middleton P, Altman DG, et al. CONSORT for reporting randomised trials in journal and conference abstracts. *Lancet* 2008, 371:281-283 [↑](#endnote-ref-1)
2. Hopewell S, Clarke M, Moher D, Wager E, Middleton P, Altman DG at al (2008) CONSORT for reporting randomized controlled trials in journal and conference abstracts: explanation and elaboration. *PLoS Med* 5(1): e20 [↑](#endnote-ref-2)
3. Ioannidis JP, Evans SJ, Gotzsche PC, O'Neill RT, Altman DG, Schulz K, Moher D. Better reporting of harms in randomized trials: an extension of the CONSORT statement. *Ann Intern Med* 2004; 141(10):781-788. [↑](#endnote-ref-3)
