# Supplementary material for: The physical activity at work (PAW) study: a cluster randomised trial of a multicomponent short-break intervention to reduce sitting time and increase physical activity among office workers in Thailand
Source: Lancet Reg Health Southeast Asia. 2022 Oct 19;8:100086. doi: 10.1016/j.lansea.2022.100086 (PMC10305858; doi:10.1016/j.lansea.2022.100086)
Supplement: Supplementary file 2 [file mmc2.docx]

**Appendix**

**Figure S1.** Participation in movement breaks in the intervention group


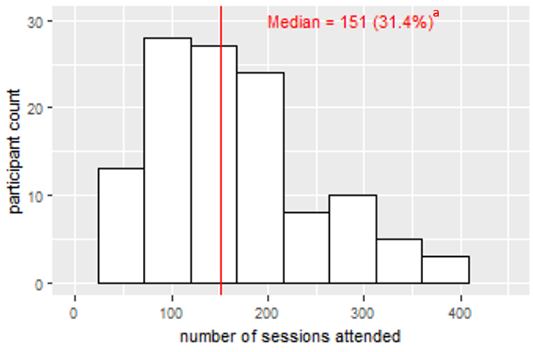


^a^ 31.4% refers to the median participation percentage out of the maximum 480 movement breaks during the 6-month intervention period

| **Table S1.** Baseline and 6-month ActiGraph™ measurements | | | | | | | |
| --- | --- | --- | --- | --- | --- | --- | --- |
|  |  | Overall | | Control | | Intervention | |
|  |  | N | Mean (SD) | N | Mean (SD) | N | Mean (SD) |
| Number of valid days (days) | Baseline | 277 | 5.3 (1.0) | 139 | 5.2 (0.9) | 138 | 5.2 (0.9) |
|  | 6-month | 247 | 5.3 (1.2) | 125 | 5.1 (0.9) | 122 | 5.6 (1.3) |
| Waking hour ^a^  wear time (h/ day) | Baseline | 277 | 13.9 (2.4) | 139 | 13.9 (2.4) | 138 | 13.8 (2.4) |
|  | 6-month | 247 | 13.8 (2.7) | 125 | 14.0 (2.4) | 122 | 13.7 (2.9) |
| Work hour ^a^  wear time (h/ day) | Baseline | 277 | 7.9 (0.2) | 139 | 7.9 (0.2) | 138 | 8.0 (0.2) |
|  | 6-month | 247 | 7.9 (0.2) | 125 | 7.9 (0.3) | 122 | 7.9 (0.2) |
| ^a^ Waking hours and work hours data obtained from participants’ daily log | | | | | | | |

| **Table S2.** Primary (time spent in sedentary behaviour) outcome with the imputation of missing values | | | | | | |
| --- | --- | --- | --- | --- | --- | --- |
|  | 6-month, Mean (SD) | | | Mean difference  (Intervention – control) | | |
| Outcome | Control  (n = 142) | Intervention  (n = 140) | | β (95% CI) ^a^ | P-value | ICC |
| Waking hours ^b^ |  |  |  |  |  |  |
| Sedentary behaviour, min | 499  (111) | 478  (128) | | -26.1  (-66.9 – 14.7) | 0.19 | 0.04 |
| Working hours ^b^ |  |  |  |  |  |  |
| Sedentary behaviour, min | 276  (49.8) | 272  (51.7) | | -4.75  (-24.5 – 15.0) | 0.62 | 0.08 |
| ^a^ Linear mixed-effect model (unadjusted), accounting for office cluster as a random effect variable  ^b^ Waking hours and working hours data obtained from participants’ daily log | | | | | | |

| **Table S3.** Tertiary outcomes | | | | | | |
| --- | --- | --- | --- | --- | --- | --- |
|  | 6-month,  Mean (SD) | | Mean difference  (Intervention – control) | | Adjusted Mean difference  (Intervention – control) | |
| Outcome | Control  (n = 125) | Intervention  (n = 122) | β (95% CI) ^a^ | P-value | β (95% CI) ^b^ | P-value |
| Percentage reduced work productivity | 21.8  (25.3) | 21.6  (27.0) | 0.25  (-8.53 – 9.02) | 0.95 | -0.37  (-9.20 – 8.47) | 0.93 |
| Musculoskeletal ^c^ complaints |  |  | Odds ratio ^c^  (Intervention – control) | | Adjusted Odds ratio ^c^  (Intervention – control) | |
| Neck pain  (count, percent) | 60  (48%) | 74  (61%) | 0.74  (0.34 – 1.61) | 0.45 | 0.77  (0.33 – 1.83) | 0.56 |
| Lower back pain  (count, percent) | 45  (36%) | 40  (33%) | 0.87  (0.51 – 1.47) | 0.60 | 0.82  (0.46 – 1.44) | 0.48 |
| ^a^ Linear mixed-effect model (unadjusted), accounting for office cluster as a random effect variable  ^b^ Linear mixed-effect model, adjusted for the respective baseline covariate  ^c^ Odds ratio for dichotomised outcomes | | | | | | |
